# The national determinants of deforestation in sub-Saharan Africa

**DOI:** 10.1098/rstb.2012.0405

**Published:** 2013-09-05

**Authors:** Thomas K. Rudel

**Affiliations:** Department of Human Ecology, Rutgers, The State University of New Jersey, 55 Dudley Road, New Brunswick, NJ 08901, USA

**Keywords:** deforestation, dry forests, Dutch disease, sub-Saharan Africa

## Abstract

For decades, the dynamics of tropical deforestation in sub-Saharan Africa (SSA) have defied easy explanation. The rates of deforestation have been lower than elsewhere in the tropics, and the driving forces evident in other places, government new land settlement schemes and industrialized agriculture, have largely been absent in SSA. The context and causes for African deforestation become clearer through an analysis of new, national-level data on forest cover change for SSA countries for the 2000–2005 period. The recent dynamic in SSA varies from dry to wet biomes. Deforestation occurred at faster rates in nations with predominantly dry forests. The wetter Congo basin countries had lower rates of deforestation, in part because tax receipts from oil and mineral industries in this region spurred rural to urban migration, declines in agriculture and increased imports of cereals from abroad. In this respect, the Congo basin countries may be experiencing an oil and mineral fuelled forest transition. Small farmers play a more important role in African deforestation than they do in southeast Asia and Latin America, in part because small-scale agriculture remains one of the few livelihoods open to rural peoples.

## 1. Introduction

Over the past 10 years, a measure of consensus has developed among analysts about the rates and drivers of tropical deforestation. The rates of deforestation appear to have continued at relatively high rates until 2005 when declines began [1]. Between the late 1970s and the early 1990s, the primary drivers of deforestation shifted from state sponsored new land settlement schemes to highly capitalized planters, agri-businesses, logging firms and cattle ranchers [2]. These historical generalizations came from empirical research on deforestation's drivers in Latin America and southeast Asia. Significantly, neither the rates of deforestation nor the forces driving it in sub-Saharan Africa (SSA) conform to these patterns. Rates of deforestation in SSA have remained much lower than either in Latin America or southeast Asia, around 0.049% per year rather than the 0.250% in Latin America and the 0.189% in southeast Asia during the 2000–2005 period [3].^1^ The drivers for deforestation also appear to be different. For decades, observers have identified rural population growth as the chief driver of deforestation in SSA [4–6]. Some of these patterns persist. The clearing of land for tropical food crops (cassava, yams) driven by the growing demands of expanding populations may explain most recent African deforestation in countries with tropical moist forests [7]. At the same time, rapid growth in urban SSA populations suggests recent shifts in the drivers of SSA deforestation. The long divergent rates of SSA deforestation, coupled with recent changes in settlement patterns and forest use, argue for an updated analysis of deforestation patterns in SSA. To this end, this paper uses newly available, national-level data on changes in African forest cover to examine the drivers of SSA deforestation during the 2000–2005 period.

The divergence between SSA and other regions in their patterns of deforestation should not be surprising given the exceptional human ecology of SSA. Three salient regional features are immediately apparent. First, the human population of SSA has long been much poorer and more rural in its distribution than the populations of Latin America and Asia. Second, relative to tropical Asia and Latin America, tropical Africa has a more arid climate [8] with seasonally dry woodlands (dry forests, thickets and other shrublands) that exceed tropical moist forests in extent [9]. Finally, five of the six Congo basin countries that contain almost all of the continent's humid forests have large extractive industries (oil and mineral) whose political economy may have had important indirect effects on forest cover. These three regional characteristics, in interaction, may begin to explain SSA's historically distinctive regional pattern of deforestation.

The following pages present this argument in a detailed way, beginning with arguments about the dynamics of national political economies that might explain long established patterns of African deforestation as well as recent shifts in those patterns. The article then describes the data that make it possible to assess, at least in part, the accuracy of this explanation. The following section presents the results of the quantitative analyses. The next section discusses how the quantitative analyses, when viewed within the human ecological context of SSA, account for recent patterns among the country-level drivers of deforestation. The paper concludes with a description of important unanswered questions about deforestation's drivers in SSA.

## 2. Why so different?: a historical argument for African exceptionalism in rainforest destruction

When natural scientists first became aware of the rapid rates of forest destruction in the tropics, many of them concluded that expanding populations of shifting cultivators had driven the rapid rates of forest decline [6]. By this logic rates of forest destruction and human population growth would vary over relatively small distances, with rapid population growth in one place contributing to lots of local deforestation while in another, not so distant, place slow population growth leads to little deforestation. For this reason, observers expected to find a close association between population growth and rates of forest destruction [6]. A look at variations in regional patterns of land cover in the Congo River basin confirms a version of this historical account of forest losses. The most densely populated regions of the country, the Bas-Congo and the eastern frontier region, contain the fewest remaining forests. Presumably, they gradually disappeared over time through repeated shifting cultivation of the same tracts of land. Corridors of cleared land emerged along major transportation axes, but the most prominent ones occurred along rivers, in part because governments, embroiled in political turmoil, could not maintain overland routes for truck traffic [10]. The absence of this state-led infrastructure effect, so evident elsewhere, may begin to explain the persistently lower deforestation rates in Central Africa.

Significant changes in political economies during the past four decades may have begun to disrupt this rural population growth-driven pattern of deforestation in the SSA states, replacing it with a more urban focused pattern of forest losses. The political and economic changes stem in large part from the increased extraction of oil and minerals from African deposits. The discovery of the deposits triggered economic booms in the extractive sectors of economies. The sectoral booms set off cascades of economic side effects that may have retarded agricultural expansion, accelerated urban expansion and probably slowed the destruction of tropical forests in remote rural places. This theoretical line of reasoning, referred to as ‘Dutch disease’, goes as follows [11,12].^2^ The rapid increase in the sale of the newly discovered commodity (oil) creates demands for labour in the booming sector which raises the price of labour throughout the country and creates problems for labour intensive enterprises such as agriculture. The high wages in the booming sector attract workers from these enterprises, creating labour scarcities or raising wages to the point where farmers can no longer profit from their work. In this context, farmers may choose to abandon agriculture. At the same time, the national currency may appreciate in value because, for example, overseas purchasers want the currency in order to purchase the extracted oil. If the agricultural sector is producing a crop for sale overseas, such as cacao, the appreciating currency (or the tie of a local currency to a hard currency, the Euro in the case of the Central African economies) makes their product more expensive relative to the same crop grown in a country with a depreciating currency, so agricultural producers from the oil producing countries would lose market share. The strong currency also makes it easier to import grains from overseas and substitute these foodstuffs for locally produced foods. For all of these reasons, a booming extractive sector would discourage agricultural expansion into forests [11,13].

Of course what appears to work in theory may not work in practice. In the case of the offshore oil deposits found in four of the six Congo Basin Forest Initiative countries, the booms generated relatively little demand for local labour, but they did lead to large increases in government receipts from royalty agreements. The increase in government revenues touched off booms in construction spending and government staffing in urban centres. The increase in urban jobs accelerated urban population growth and, by pulling rural people off of the land, it should have discouraged agricultural expansion, so, in theory, the booms would have had an overall forest preserving effect.

Consistent with this pattern, societies in SSA had the highest rates of urban population growth, 3.71%, in the world between 2000 and 2005 [12]. With this urban focused pattern of population growth, forest losses would occur, but they would not be associated with rural population growth as may have been the case during the decades following World War II. Instead, the forest losses would occur primarily in peri-urban regions and in transportation corridors between cities. The growing concentration of people in urban areas would encourage people to make increasing use of charcoal for cooking. Cutover districts outside of urban areas would grow in size as local residents harvest woody vegetation to make charcoal for shipment to urban markets [14,15]. The growth in urban population would also encourage increases in the shifting cultivation of staple crops in peri-urban zones, a pattern that would be consistent with the reported association between recent deforestation and growth in areas cultivated for staple crops [7]. Bolstered by a strong currency, merchants might begin importing more cereal crops from overseas to feed urban populations, thereby depressing agricultural expansion in the oil producing countries. Together, these effects would reduce deforestation rates, creating a Dutch disease-induced forest transition [6].

These theoretical expectations would of course play out in a larger regional agro-ecology marked by extensive areas that are not suitable for agriculture because they receive so little rain [16]. This circumstance would increase human pressure on the wet and semi-arid zones that receive enough rain to grow crops, so higher rates of deforestation might occur in countries with relatively small humid zones that contain both croplands and forests.

In theory then, the recent growth in extractive industries would contribute indirectly to urban population growth and retard agricultural expansion, especially in the humid forest zones where the extractive industries have been especially dominant. The resulting patterns of forest loss should have an urban focus and be more prevalent in countries with dry forests. Recently published, remote-sensing-derived estimates for national changes in sub-Saharan forest cover provide an opportunity to put these expectations to an empirical test.

## 3. Data and methods

Over the past 25 years, scientists have conducted hundreds of studies, similar to this one, of variations across nations in rates of deforestation. The units of analyses in these studies follow the political boundaries of states rather than the ecological boundaries of watersheds or forests. The use of political units of analysis acknowledges that, because human behaviours drive deforestation, human variability from nation to nation may provide crucial evidence about the configuration of driving forces that generates forest clearing. The use of the nation state as a unit of analysis for studying deforestation may seem too coarse grained to portray and analyse the household decision-making that drives deforestation. Certainly, micro-focused studies of deforestation have value, but so do larger scale studies, largely because they incorporate national-level variables such as large-scale tax receipts from petroleum production into our understanding of the drivers of landscape changes. Coarse-grained studies similar to this one also make it possible to incorporate regional differences in landscapes, between humid and dry tropical forests for example, into analyses.

All of the deforestation studies prior to 2000 used unreliable measures of forest losses gathered using different procedures from country to country [17]. The availability of multi-scalar, remote-sensing-based measures of forest loss after 2000 eliminated this source of measurement error. The multi-scalar measures were developed through a two-step procedure in which lower resolution MODIS (moderate resolution imaging spectroradiometer) imagery was used to classify biomes into areas with different degrees of land cover change and then higher resolution Landsat imagery was used to generate estimates of deforestation for each area. Extrapolations then generated estimates of forest loss for larger areas [18]. The data first became available for the 2000–2005 period and their superiority over previous measures explains why this study focuses on forest cover change during the 2000–2005 period. The same data make it possible to estimate the carbon stock density of forests in countries [19,20]. These estimates include above- and below-ground carbon, and they vary with climate. Dry, open forests typically have much lower carbon densities than do humid, closed canopy forests. These measures of carbon stocks are particularly important for studying the drivers of deforestation in SSA, because they provide a way of distinguishing between processes involving denser, more humid forests and those involving the less dense, dry woodland forests so prevalent in African settings.^3^

While the MODIS data represent a distinct improvement over previous national-level deforestation data, the coarse-grained resolution of the MODIS data could produce erroneous national estimates, especially in the smaller- to medium-sized countries with fewer pixels. Acknowledging this potential source of error, the conceivable salience of macro-scale determinants of deforestation in SSA argue for doing an analysis of the cross-national variations in the MODIS deforestation data. The measures for the variables in these multivariate analyses and the sources for these data are listed in table 1.

**Table 1.** Measures for variables and sources for data.

variables and measures	sources
dependent variable (in table 3)	
*Deforestation rate*. MODIS imagery for entire areas of interest combined with Landsat Thematic Mapper images for selected areas generated the estimates of forest losses from 2000 to 2005. The losses were then divided by forest area in 2000 in order to obtain the deforestation rate	Hansen *et al*. [18]
independent variables (in table 3)	
*Forest stock density*. This measure is derived from estimates of biomass carbon stocks	Saatchi *et al*. [21]
*Imports of cereals* per capita, *2000*. Tons of cereals imported in 2000/population of the nation, 2000	Earthtrends (http://earthtrends.wri.org/)
*Oil and gas receipts as a proportion of all receipts from exports, 2000*. The proportion of total export earnings from fuels and minerals for a country in 2000	UNCTADstat (http://unctad.org/en/Pages/Statistics.aspx)
*Proportion of the population living in an urban area, 2000*	United Nations Population Division of the Department of Economic and Social Affairs, 2007, world population prospects: the 2006 revision and world urbanization prospects: the 2005 revision (http://esa.un.org/unpp)
*Proportion of lands unsuitable for agriculture*. Percentage of the national land area that is considered unsuitable for agricultural production	FAO–IIASA Project on Global Agro-ecological Zones (http://www.iiasa.ac.at/research/LUC/GAEZ/index.htm)
*Population growth, 1990s*. (population, 2000 – population, 1990)/rural population, 1990	Population Division, Department of Social and Economic Affairs, United Nations.
other variables (in table 2)	
*Gross domestic product*, per capita, *2000*	Penn World Tables (http://pwt.econ.upenn.edu/php_site/pwt70/pwt70_form.php)
*population, 1990 and 2000*	

The analysis presented in table 3 regresses a series of independent variables that represent different aspects of the causal arguments presented above on variations across nations in deforestation rates. In effect, the equation represents an inventory of causes. The independent variables all represent conditions before 2000, whereas the dependent variable represents processes occurring during the 2000–2005 period, so the equations do not exhibit simultaneity biases. The univariate Moran's I for the deforestation variable was 0.252, so some spatial autocorrelation did characterize the deforestation data. For this reason, the analyses in table 3 use spatially lagged regression models that allow for deforestation in one spot during one period to create deforestation in adjacent spots in subsequent periods.^4^ The dataset for these analyses is available in the electronic supplementary material.

## 4. Findings

Table 2 outlines major subregional differences in land cover and societal trends in SSA. It distinguishes between the nations with more humid forests in Central Africa and the surrounding nations in eastern, western and southern Africa that contain mostly dry forests. The Democratic Republic of the Congo (DRC) merits its own entry because it is so large relative to the other countries and represents an unusual combination of extremely poor people living amid mineral riches and extensive rainforests. As indicated by the differences in carbon stock densities (row 1, table 2), the South, East and West African nations contain a preponderance of the sparser, dry forests. They exhibited higher deforestation rates during the 2000–2005 period, contained larger areas of lands unsuitable for agriculture (too arid), lower rates of population growth and less urbanized populations. Their economies also depended less on receipts for oil and gas than did the economies of Central Africa. While the Central African nations (row 2, table 2) contained a preponderance of the continent's humid tropical forests (see the carbon stock densities), elevated receipts from mineral trade, more urbanized populations and lower deforestation rates than elsewhere in SSA, the DRC (row 3, table 2) deviated from the Central African pattern in important ways, in particular in the continued poverty of its population.

**Table 2.** The dynamics of deforestation in SSA, 2000–2005: subregional patterns. The significance tests in the table pertain to mean differences between the Central African countries and South, West and East African countries in rows 1 and 2.

	(1) oil/gas receipts as % of all export $	(2) GDP *per capita*, 2000 ($)	(3) % of people living in cities, 2000	(4) population growth in the 1990s (%)	(5) % of lands unsuitable for agriculture	(6) forest carbon stock density (Mg of C ha^−1^)	(7) forest losses (% per year)
(1) East, West and South African countries	19.8**	1423**	31.9**	26.7*	52*	50**	0.617*
(2) Central African (Congo Basin) countries, excluding DRC	61.4	4292	53.0	41.9	31	138	0.155
(3) DRC	21.0	124	29.8	37.9	30	128	0.121

*p < 0.10, **p < 0.01.

**Figure 1.**
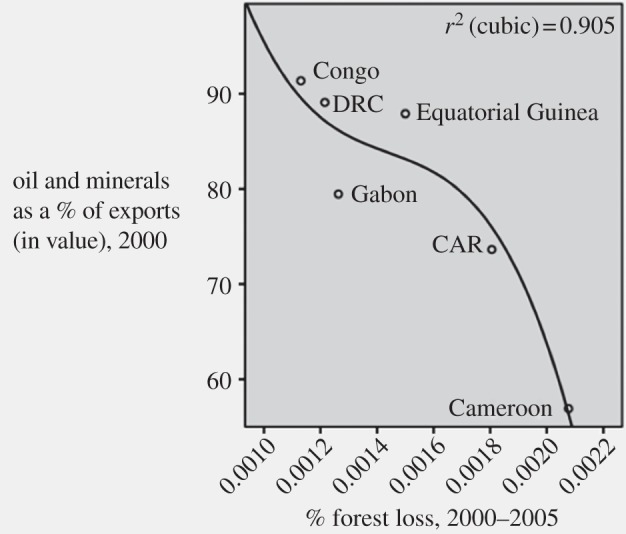
Oil and mineral exports and deforestation in the Congo Basin. CAR, Central African Republic; DRC, Democratic Republic of the Congo.

The analyses reported in figure 1 and in table 3 provide evidence that bears on the hypothesis that accelerated urbanization rates, spurred in a significant number of countries by oil and mineral booms, has driven SSA deforestation during the post millennium period. The scatterplot in figure 1 shows a very high degree of association between dependence on oil and mineral exports and low deforestation rates (*r*^2^ = −0.900, *p* = 0.014), as Dutch disease theory would predict.

**Table 3.** National determinants of deforestation in SSA, 2000–2005: spatially lagged regressions. N.B. These multivariate analyses regress a series of independent variables on variations across countries in deforestation rates. The first number in a row is the unstandardized regression coefficient for that independent variable. The number in parentheses is the error term for that coefficient.

	all countries	arid countries (countries in SSA outside Congo Basin)
independent variables		
proportion of lands that are unsuitable for agriculture	0.010*** (0.001)	−0.135*** (0.040)
carbon stock density in forests	−0.040*** (0.010)	1.247*** (0.330)
proportion of the population living in urban areas, 2000	0.016*** (0.003)	
oil and gas receipts as % of exports	−2.94* (1.57)	
cereal imports *per capita*	−0.046** (0.014)	
population growth 1990s (% per annum)		0.202* (0.079)
* r*^2^	0.688	0.670
* n*	38	32

*p < 0.10, **p < 0.01, ***p < 0.001.

The findings from the spatial regression analyses reported in table 3 underline the differences between deforestation processes in the dense, humid forest countries of the Congo basin and the predominantly dry forest countries elsewhere in SSA. Deforestation rates tend to be higher in countries with little potential for rainfed agriculture, less dense forests, small oil–mineral sectors, little reliance on imported cereals and more urbanized populations. Similar analyses of only countries with predominantly dry forests finds more rapid deforestation in dry forest countries with more potential for rainfed agriculture, more dense forests and rapid population growth.

## 5. Discussion

The results from the multivariate analyses reported in table 3 underscore several points about the national-level drivers of deforestation in SSA. The most extensive clearing occurred in dry forest areas, so the countries with less dense forests and lots of arid lands unsuitable for agriculture experienced higher rates of deforestation. Consistent with Dutch disease theory, a large oil and gas sector and the large-scale importing of cereals tended to depress deforestation rates (column 1, table 3), but urbanization accelerated forest clearing, perhaps because it generated increased demand for agricultural products. In the arid environments of the countries outside the Congo basin (column 2, table 3), the countries with the denser forests and most potential for rainfed agriculture saw the most deforestation. Population growth, wherever it occurred in these countries spurred deforestation.

Although all of these associations are at a large-scale, they fit the patterns evident in more micro-scale analyses of forest cover change in the SSA. In ‘State and the evolution of African rainforests between 1990 and 2010’ in this issue [6], the authors find subregional differences in deforestation rates consistent with the Dutch disease hypothesis, with the lowest rates of deforestation, as indicated in figure 1, occurring in oil- and mineral-dependent societies. The Central African societies, not coincidentally, have the lowest rural population densities. While these low densities no doubt represent historical legacies of earlier eras, the Dutch disease dynamic with the impetus that it gives to urbanization would encourage rural to urban migration and depress rural population densities. Accelerated urbanization would also explain the emerging spatial form of deforestation evident in this study, more pronounced deforestation around cities and along transportation corridors between cities [6]. The findings about urbanization contradict the Dutch disease hypothesis and suggest that at least in some countries urbanized populations may spur deforestation through their growing demand for agricultural products. This line of reasoning has recently found support in other studies of the cross-national drivers of deforestation [3,7].

Deforestation in the DRC departs from the regional pattern in its trend lines but resembles the regional pattern in its drivers. Unlike other countries in the Congo Basin Partnership, deforestation in the DRC appears to have increased significantly from 1990–2000 to 2000–2005 [22]. Political turmoil has prevented significant economic growth and increases in income for decades in the DRC. At the same time, urban population growth, as in other SSA countries, has continued at high rates (+4.15% per year between 2000 and 2005). In this context, smallholders' reliance on the shifting cultivation of cash crops has, if anything, increased, so DRC deforestation may be becoming more urban focused at the same time that its magnitude has increased.

More generally, the DRC case exposes the hazards of continental-scale generalizations. The dynamics of deforestation in the DRC appear to differ significantly from the dynamics in the other Congo Basin countries where humid forests predominate. These differences in humid forest zone deforestation dynamics need to be viewed in the context of large differences in deforestation dynamics between countries with predominantly humid forests, such as Gabon and Congo, on the one hand and countries with predominantly dry forests, such as Tanzania and Zambia, on the other hand. While there are common elements that, together, compose a continental pattern of deforestation, urbanization being one important factor, no one country fully exemplifies the larger pattern apparent in this analysis.

## 6. Conclusion: Whither African exceptionalism in deforestation?

Do the patterns described above attest to a continuation into the twenty-first century of African exceptionalism in forest destruction? The analysis carried out here suggests that crucial differences in the context rather than in the dynamics of deforestation explain the differences in deforestation processes between SSA and other regions. The people are poorer, the extractive sectors of economies are larger, and the climate is more arid.

Acknowledging these differences, some deforestation pressures seem common to all regions. Growth in urban populations encouraged tropical deforestation in southeast Asia and Latin America early in the twenty-first century [3], and the shifting patterns of deforestation in SSA suggest a similar process at work in SSA. Recent studies [6] and the analysis here suggest that growth in urban populations has spurred agricultural expansion in places close to cities or with good transportation links to cities. For example, the growth of the Kinshasa urban agglomeration in the DRC led to an expansion in smallholder agriculture some 400 km to the north of the city along the Congo where the river makes it relatively easy to ship foodstuffs downstream to the city [2,22,23].

Poverty explains one important difference between African deforestation and Asian–Latin American deforestation. While large plantations, farms and ranches have become the primary agents for tropical deforestation in southeast Asia and South America, smallholders continue to play an important role in driving African agricultural expansion and forest losses. The continuing salience of smallholders in African agricultural expansion reflects the poverty of the people in the region and in particular the urbanization of societies without industrialization. The absence of well-remunerated non-farm jobs in cities and elsewhere makes the continued exploitation of the forests through shifting cultivation and charcoal production a more attractive prospect for rural people [10].

The rapid growth of SSA cities reflects in part the economic booms brought by increased receipts from extractive enterprises. Food imports increased, especially in oil-based economies with strong currencies, and, as evident in table 3, these imports discouraged agricultural expansion, especially in remote agricultural zones. In this respect, the salience of extractive industries in African economies has contributed, through its contribution to accelerated urbanization, to reduced rates of deforestation.

Some subregional differences raise important questions for further research. Among the most pressing questions raised by this analysis involves the higher rates of deforestation occurring outside of the humid Central African forests. Recent reports show a concentration of fires in the dry forest regions [10], perhaps with some connection to agricultural expansion. Ecologists have long argued [24] that fire plays an important role in the destruction of dry forests in the tropics. The losses of biodiversity as well as the additional greenhouse gas emissions that occur with the burning of dry forests would seem to make a better understanding of the dynamics of dry forest destruction an important item in the research agendas of land change scientists in Africa.

## Endnotes

1 The deforestation rates in this study were calculated as old growth forest lands lost as a percentage of the total tropical biome in a country, so they are lower rates than are often reported in studies of deforestation that use total tropical forest area rather than tropical biome area as the denominator in calculations [3].

2 The theory was first illustrated through an analysis of the effects of a boom in natural gas extraction in The Netherlands, so analysts have taken to referring to it as ‘Dutch disease’.

3 Degradation occurs in both humid and dry forests, and in both instances it would lower carbon stocks from the levels usually observed in undisturbed forests.

4 The six Congo Basin countries in table 2 are Cameroon, Central African Republic, the Congo, the DRC, Equatorial Guinea and Gabon. The 32 West, South and East African countries are Angola, Benin, Botswana, Burundi, Cote D'Ivoire, Chad, Ethiopia, Gambia, Ghana, Guinea, Guinea-Bissau, Kenya, Lesotho, Liberia, Madagascar, Malawi, Mali, Mozambique, Namibia, Nigeria, Rwanda, Senegal, Sierra Leone, Somalia, South Africa, Sudan, Swaziland, Tanzania, Togo, Uganda, Zambia and Zimbabwe. The two sets of countries were combined for the regression analysis presented in table 3.
